# Feasibility of heart rate monitoring in hemodialysis patients using visible imaging: a pilot study

**DOI:** 10.3389/fphys.2026.1909505

**Published:** 2026-08-20

**Authors:** Uday Debnath, Sreya Deb Srestha, Sungho Kim

**Affiliations:** Department of Electronic Engineering, Yeungnam University, Gyeongsan-si, Republic of Korea

**Keywords:** heart rate, hemodialysis patient, multi-method ensemble fusion, rPPG, visible imaging

## Abstract

**Background:**

Continuous vital signal monitoring is essential for patients undergoing hemodialysis sessions in which physiological instability, body fatigue, and cardiovascular complications may occur and require early medical interventions. However, conventional contactbased sensors such as electrocardiogram (ECG) have several limitations due to increased skin sensitivity, skin irritation, and interference with clinical procedures.

**Objective:**

This study proposes a remote photoplethysmography (rPPG) framework for continuous heart rate monitoring of stage-5 chronic kidney disease (CKD-5) patients.

**Methods:**

A pilot cohort of eight patients diagnosed with stage-5 chronic kidney disease undergoing hemodialysis sessions were monitored using synchronized RGB video and ECG reference measurements to generate 32 hours of video data. The proposed framework incorporates an automated facial landmark tracking and a dual-masking-based skin detection approach to robustly define regions-of-interest (ROI). A multimethod ensemble fusion is integrated through spectral analysis, peak detection, and harmonic product spectrum analysis for confidence-weighted final heart rate estimation.

**Results:**

The proposed framework achieved a mean absolute error (MAE) of 1.16 beats per minute (BPM) and root mean squared error (RMSE) of 1.86 BPM with a mean bias error of −0.68 BPM among all patients irrespective of lower visibility due to mask and partial occlusion.

**Conclusion:**

These findings suggest that noncontact heart rate monitoring of patients diagnosed with advanced renal diseases is feasible using visible imaging and the rPPG method. By addressing clinical variability, the proposed framework suggests the potential of remote heart rate monitoring in a controlled dialysis-unit environment.

## 1 Introduction

Chronic kidney disease (CKD) is a major global health concern and is closely associated with increased cardiovascular mortality (Matsushita et al., 2022). Dialysis patients are vulnerable to cardiac health complications, including cardiac arrhythmia and autonomic dysfunction, and require continuous physiological monitoring (Rogovoy et al., 2019). In healthcare facilities, vital signals are usually monitored using traditional contact-based sensors, such as electrocardiograms (ECGs) and pulse oximeters (Grove et al., 2023). Although these sensors provide accurate measurements, these may cause skin irritation and discomfort during prolonged uses particularly in sensitive patients with skin fragility and edema (Zhou et al., 2024). In addition, contact-based sensors restrict patient’s body movement and make them uncomfortable during continuous treatment sessions (Khatsenko et al., 2020). These limitations highlight the need for an alternative method for continuous heart rate (HR) monitoring that is less intrusive and more suitable for subjects in critical clinical conditions (Jorge et al., 2022).

Remote photoplethysmography (rPPG) has emerged as a key alternative to contact-based approaches for vital sign monitoring such as HR (Aarts et al., 2013). It measures HR signals by analyzing subtle blood volume pulse variations through light reflectance from skin regions (Almarshad et al., 2022). Most previous studies have been conducted in controlled scenarios with healthy subjects (Scardulla et al., 2023). Recent studies have shown that, rPPG-based approaches can detect cardiac arrhythmia in earlier stages, which is effective for patient monitoring in clinical care settings (Xiao et al., 2024). Despite these advancements, rPPG method exhibits low accuracy due to noise and motion artifacts in real-world conditions (Nguyen et al., 2024). Ambient lighting fluctuation and dynamic patient motion can also degrade overall signal quality with low signal-to-noise ratio (SNR) (Dasari et al., 2021). Previous state-of-the-art (SOTA) models have been evaluated on healthy subjects with minimal to no clinical complications, which may not reflect the physiological variability observed in patients undergoing critical diagnosis such as hemodialysis conditions (Zou et al., 2024). Therefore, the lack of clinical validation makes it difficult to consider rPPG as an alternative approach in conventional healthcare facilities.

This study proposes a motion robust adaptive ROI framework with multi-method ensemble fusion strategy for continuous HR monitoring. It incorporates adaptive ROI tracking, a sequence of signal preprocessing stages, and continuous system calibration to remove noise artifacts and ensure rPPG signal quality during the physiological shifts throughout dialysis session. The key contribution of the study is it evaluates the feasibility of continuous heart rate monitoring during prolonged sessions using visible imaging. Proposed framework shows higher performance metrics while validating its overall performance compared to ground-truth reference values for remote health monitoring in clinical environments.

## 2 Materials and methods

### 2.1 Experimental setup and data collection

**Figure 1:**
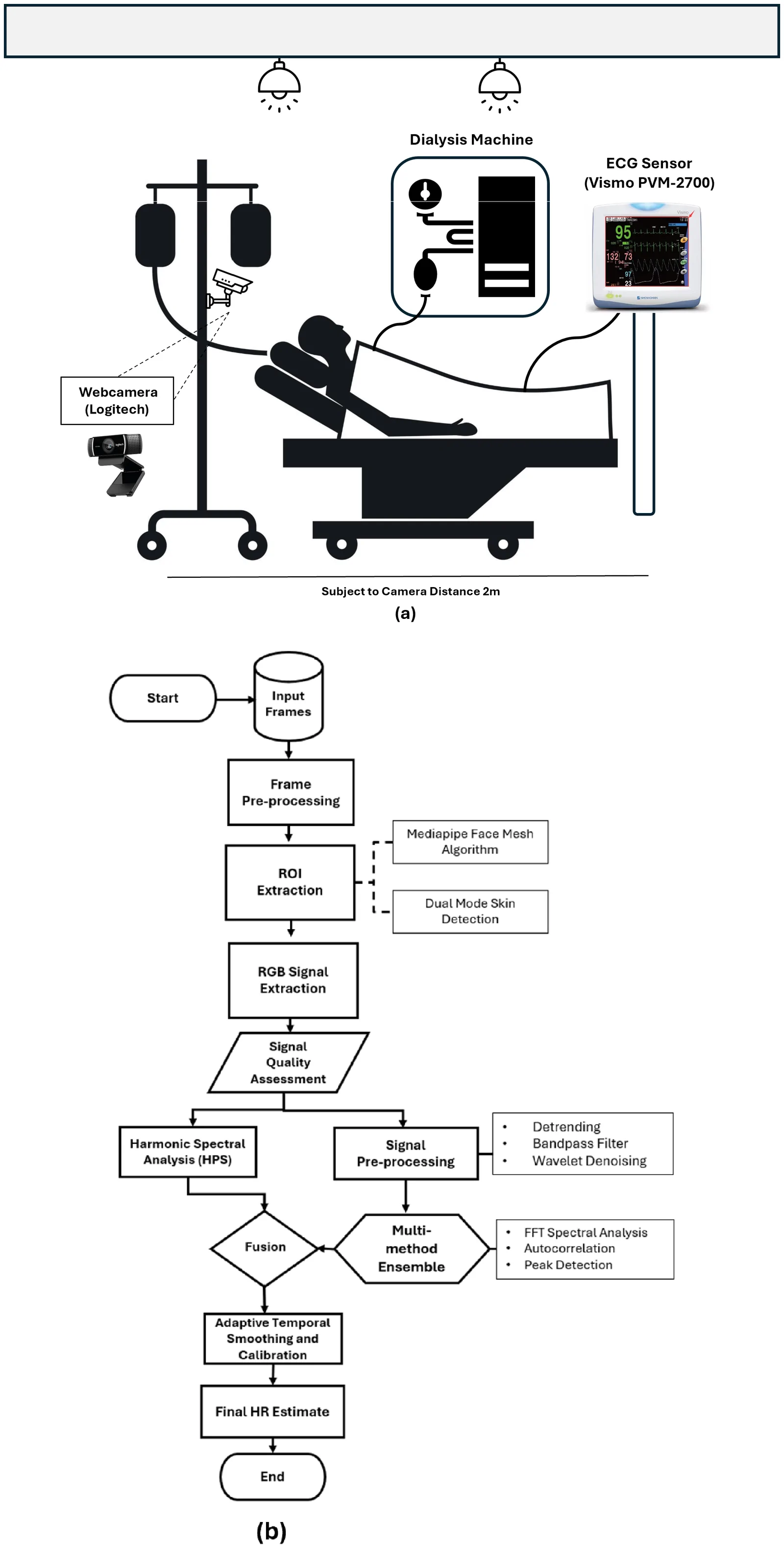
Overview of the proposed study, **(a)** experimental setup and **(b)** proposed framework.

This study collected a custom dataset from an advanced renal unit during routine hemodialysis sessions using a Logitech RGB webcam, as shown in **Figure 1a**. Video recordings were acquired under natural indoor lighting conditions, while an electrocardiogram (ECG) monitor (Vismo PVM-2700) simultaneously provided the ground-truth (GT) reference values that are stored in .xlsx files. The webcam was positioned at a fixed distance of approximately 2 meter from the patient, facing the upper body and facial region to ensure consistent visibility of facial features. Participants remained in a supine position throughout dialysis sessions without movement restrictions allowing natural head motion and postural adjustments. A pilot cohort of eight patients undergoing hemodialysis was recorded which resulted in approximately 1920 minutes (32h) of video data collected at a frame rate of 30 frames per second (fps). During postprocessing, frame indices were monitored to maintain temporal consistency between the recordings and the GT reference values without any noticeable synchronization mismatch was observed throughout the recording sessions. To establish temporal synchronization, each analysis window was aligned with the corresponding ECG measurements with 1 Hz frequency. This window-based synchronization ensured that each rPPG-derived HR estimate was compared with the temporally alligned GT reference values over the same observation period. All participants provided written informed consent, and the study protocol was approved by the Yeungnam University Institutional Review Board (Approval No. 7002016-A-2024-114).

### 2.2 Proposed methodology

The proposed framework incorporates five key modules as illustrated in **Figure 1b** including: (i) frame preprocessing and illumination normalization, (ii) facial skin segmentation and region-of-interest (ROI) extraction, (iii) temporal signal extraction and signal quality assessment, (iv) signal preprocessing and multi-method spectral analysis, and (v) confidence-weighted adaptive fusion and system calibration for final HR estimation.

### 2.3 Frame preprocessing and illumination normalization

Each video frame undergoes an initial preprocessing stage to ensure consistent color segmentation to ensure consistent frame capturing at varying lighting conditions. The ambient illumination level of each frame is assessed from the mean intensity of its grayscale representation, where the frames falling below a minimum brightness level are enhanced using the contrast-limited adaptive histogram equalization (CLAHE) (Musa et al., 2018) exclusively to the luminance channel (L) of the CIE LAB color space which is adaptive histogram equalization. Following illumination correction, a spatial smoothing method is applied using a gaussian kernel to suppress sensor noise and minor pixel-level fluctuations in Equation 1 as:

(1) $$I_{c}\;\left(x,y\right)=I\left(x,y\right)*G_{\sigma }\left(x,y\right)$$

where *I*(*x,y*) denotes the input frame and *G_σ_*(*x,y*) the Gaussian kernel with standard deviation *σ*, and ∗ denotes the convolution operation.

### 2.4 Facial skin segmentation and ROI extraction

Facial region localization and ROI tracking are performed using the MediaPipe Face Mesh framework (Lugaresi et al., 2019), which provides dense three-dimensional (3D) facial landmark estimation from input videos. It shows predefined facial landmarks that capture the geometric structure of the face and enable consistent tracking across each frames. Then, detected ROI landmarks are used to define anatomically stable regions as the forehead and cheek areas, which are less affected by motion artifacts and partial occlusion.

To further refine the sub-ROIs, skin regions are identified through a dual-mode skin segmentation module operating concurrently in the YCrCb and HSV colour spaces. Independent binary skin probability masks are derived from each color space. The resulting skin probability maps are merged and then enhanced through morphological post-processing stages to mitigate false ROI detections and unify neighboring skin areas. From the refined mask, candidate regions are extracted and evaluated via a weighted mechanism following Equation 2:

(2) $$S_{\text{confidence}}=w_{1}\cdot S_{\text{pos}}+w_{2}\cdot S_{\text{shape}}+w_{3}\cdot S_{\text{size}}$$

where, *S*_size_ denotes the relative spatial extent of each region, *S*_shape_ penalizes geometrically irregular contours inconsistent with facial anatomy, and *S*_pos_ defines physiologically relevant regions within the frame. The corresponding weights *w*_1_ = 0.30, *w*_2_ = 0.20 and *w*_3_ = 0.50 were assigned *a priori* based on physiological domain knowledge which were fixed prior to any experimental evaluation to ensure dataset independent optimization. The dominant weight assigned to region size (*w*_3_) exhibits the relationship between ROI area and pulse related signal-to-noise (SNR) ratio, whereas larger skin-pixel reduce spatial averaging noise in the extracted signal. The positional weight (*w*_1_) was set as the second largest ROI contributor the forehead which exhibit stronger pulsatile colour modulation (Huelsbusch and Blazek, 2002). The highest-ranked regions are jointly used to form the final binary ROI mask for raw signal extraction.

### 2.5 Temporal signal extraction and signal quality assessment

For each frame, pixel intensities within the ROI masks were spatially averaged to generate temporal signals using a hybrid chrominance (CHROM) and plane orthogonal to skin-tone (POS) algorithm. Heart-rate estimation was performed using an 8-s sliding window with a 1-s update interval (87.5% overlap). Windows with insufficient signal quality or missing synchronized ECG reference measurements were excluded before statistical analysis. Within each window, low-frequency drift was removed using a third-order polynomial detrending method, followed by a fourth-order zero-phase Butterworth band-pass filter. Wavelet denoising was then performed using the Daubechies-4 (db4) wavelet with three decomposition levels and soft thresholding before signal reconstruction. These parameters were selected to provide sufficient frequency resolution while maintaining continuous HR updates. These parameters were selected to balance frequency resolution and temporal responsiveness. The relatively long window provides sufficient cardiac cycles for stable spectral estimation, while the 1-second processing step ensures clinically responsive HR update rate. HR estimates were generated for every analysis window and compared with the corresponding synchronized GT reference measurements. The sliding-window aggregation of per-frame spatially averaged RGB channel intensities provide improved spectral frequency resolution by enhancing the separation of cardiac-related signal from noise components. By projecting temporally normalized RGB channels, the CHROM method (de Haan and Jeanne, 2013) detects pulse signal that effectively eliminates specular reflections while maintaining dynamic signal modulation. Then, a complementary POS method (Wang et al., 2017) attenuates motion-induced physiological shifts. Prior to spectral analysis, a Signal Quality Index (SQI) was computed for each analysis window using welch’s power spectral density (PSD) (Liu et al., 2023) to quantify signal reliability. The cardiac frequency band was defined as 0.75–2.40 Hz (45–140 BPM), and the SQI combined the normalized cardiac-band power and normalized signal-to-noise ratio (SNR), where the SNR was calculated following Equation 3 as.

(3) $$SNR_{\text{dB}}=10\underset{10}{\log}\;(\frac{P_{HR}}{P_{total}-P_{HR}}),$$

where *P*_HR_ and *P*_total_ denote the cardiac-band and total spectral power, respectively. The overall SQI is then computed following Equation 4 as:

(4) $$SQI=0.5\left(\frac{P_{HR}}{P_{total}}\right)+0.5\min\;\left(1,\max\;\left(0,\frac{SNR_{dB}}{15}\right)\right),$$

where the SQI is bounded within [0,1]. The corresponding weight assigned to *i* − *th* analysis window is then defined by Equation 5:

(5) $$c_{i}=SQI_{i},$$

such that windows with higher spectral quality contribute more strongly during confidence-weighted fusion and adaptive temporal refinement, whereas lower-quality windows are proportionally downweighted.

### 2.6 Signal preprocessing and multi-method spectral analysis

The raw signal within each analysis window is conditioned through an adaptive signal preprocessing pipeline. First, a polynomial detrending is implemented to remove low-frequency drift induced by gradual head movements (Tarvainen et al., 2002). Second, a zero-phase fourth-order butterworth bandpass filter (Elgendi, 2012) isolates the cardiac frequency band while suppressing respiratory and motion-induced noise artifacts without introducing phase distortion. Third, a wavelet-based denoising module is employed to further reduce high-frequency noise by preserving the morphological characteristics of the physiological signal. Then, a multi-method spectral analysis methods are implemented to the raw signal to improve robustness of the rPPG signal against artifacts inherent to any single spectral analysis. First, a zero-padded, Hanning-windowed discrete fourier transform identifies the dominant spectral component within the cardiac band within frequency resolution refined via parabolic interpolation on the three-point neighborhood of the spectral peak (Schäfer and Vagedes, 2013). Second, an autocorrelation-based peak detection algorithm derives HR from the median inter-beat interval (IBI) of physiologically relevant signal components by removing noise outliers through inter-quartile range filtering (Charlton et al., 2018). Third, the harmonic product spectrum (HPS) analysis reinforces the fundamental cardiac frequency by iteratively multiplying integer-downsampled copies of the magnitude spectrum where the primary spectral peak is partially obscured by motion harmonics (Yi et al., 2022).

### 2.7 Confidence-weighted adaptive fusion for HR estimation

In this stage, multiple HR estimation strategies, including spectral analysis, peak interval estimation, and harmonic product spectrum (HPS) analysis, were integrated using a consistency-aware confidence-weighted fusion framework. Each estimator generated an independent HR estimate along with a corresponding confidence score derived from signal quality and estimator reliability. When each estimation methods exhibited a high degree of mutual agreement determined by the inter-estimator standard deviation, the fused HR was computed as a normalized confidence-weighted average following Equation 6 as:

(6) $$HR_{\text{fused}}=\frac{\sum_{i}c_{i}f_{i}}{\sum_{i}c_{i}}$$

where, *f_i_* is an estimated value for each estimation methods along with an associated confidence score *c_i_* that is subsequently propagated throughout the fusion stage. The fused HR estimate was subsequently refined through a multi-stage temporal stabilization process. First, a confidence-guided outlier suppression was applied to reduce abrupt fluctuations and maintain temporal continuity of the pulse-related waveform (Abdulsadig and Rodriguez-Villegas, 2024). Next, an adaptive Kalman filtering framework modeled the temporal evolution of the cardiovascular signal. The influence of each measurement was dynamically adjusted according to its estimated signal quality, enabling reliable observations to contribute more strongly while attenuating noisy measurements. To further improve temporal consistency, a quality-adaptive exponential smoothing stage was employed, with the smoothing factor automatically adjusted according to the overall confidence of the HR estimate. Finally, a subject-specific continuous calibration mechanism was performed using synchronized GT reference measurements to compensate for systematic bias. The calibration offset was initialized during a short adaptation period and updated using synchronized ECG reference measurements during the calibration stage gradually throughout continuous monitoring to account for slow physiological variations. The resulting HR estimate was constrained within a physiologically plausible range of 45–140 BPM to ensure robustness and clinical applicability.

## 3 Results

To quantitatively evaluate the performance metrics of the proposed framework, the mean absolute error (MAE) and root mean squared error (RMSE) are computed over N number of samples following Equations 7–9 as:

(7) $$\text{MAE}=\frac{1}{N}\sum_{i=1}^{N}\left|y_{i}-{\hat{y}}_{i}\right|,$$

(8) $$\text{RMSE}=\sqrt{\frac{1}{N}\sum_{i=1}^{N}{\left(y_{i}-{\hat{y}}_{i}\right)}^{2}},$$

(9) $$\text{Bias}\left(\text{Mean Error}\right)=\frac{1}{N}\sum_{i=1}^{N}\left({\hat{y}}_{i}-y_{i}\right),$$

where, ${\hat{y}}_{i}$ denotes the predicted HR values and *y_i_* denotes the GT values. **Table 1** summarizes the ablation study of the multi-method fusion and performance comparing of the proposed framework across individual patients where it achieved mean absolute error (MAE) of 1.16 beats per minute (BPM) and root mean squared error (RMSE) of 1.86 beats per minute (BPM) with mean bias error of −0.68 BPM. The proposed framework achieved consistently low error rates across all patients indicating reliable HR estimation across all the subjects.

**Table 1:** Ablation study and performance comparison of the proposed framework.

Method	MAE (BPM)	RMSE (BPM)
*Ablation Study*		
Peak Interval	2.86	3.69
Spectral Analysis	2.06	2.70
Harmonic Product Spectrum (HPS)	1.37	1.89
Proposed Framework	1.16	1.86
Method	MAE (BPM)	RMSE (BPM)
Performance Comparison		
Green (Verkruysse et al., 2008)	11.32	14.19
PBV (de Haan and van Leest, 2014)	11.52	13.40
CHROM (de Haan and Jeanne, 2013)	10.73	13.81
ICA (Poh et al., 2011)	8.90	11.04
POS (Wang et al., 2017)	7.50	9.32
Proposed (Ours)	1.16	1.86

### 3.1 ROI selection and signal extraction

**Figure 2:**
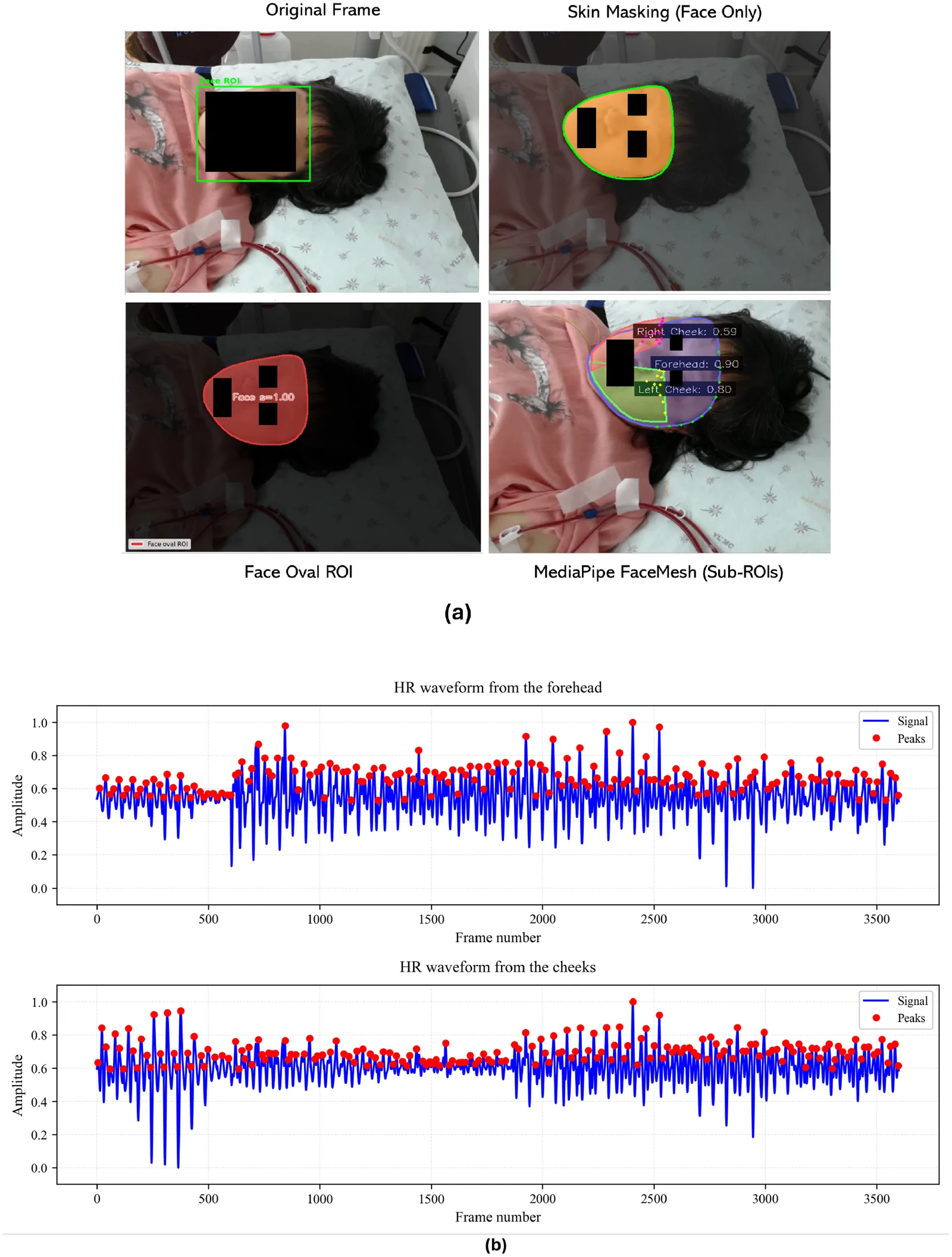
Face detection and peak detection **(a)** ROI detection and signal extraction **(b)** peak detection from sub ROIs.

**Figure 2a** shows the sequential process of ROI selection and signal extraction from facial video frames. The initial step involves face localization, where a bounding region is identified to constrain further processing to the relevant facial region. It reduces the influence of background noise and non-physiological scenes. Then, a dual-mode skin segmentation is implemented to isolate the facial skin regions and excluding background noise such as hair, resulting in a spatially coherent skin mask. The resulting mask demonstrates a high spatial coverage of the key facial area ensuring that sufficient pixel information is retained for further raw signal analysis. Then, detected facial regions are refined into a unified ROI, which serves as the basis for physiological signal extraction. To further improve signal stability, the framework highlights the facial region into anatomically consistent sub-regions, including the forehead and both cheek areas using the MediaPipe Face Mesh algorithm (Lugaresi et al., 2019). Each region is then assigned a signal quality weight reflecting its relative reliability and sensitivity to motion. This weighted configuration ensures that the extracted signals originate from physiologically meaningful and robust regions.

Face visibility shows 98.3% accuracy throughout the session with successful ROI detection from each input frames resulting in an ROI loss rate of 1.7%. Motion visibility score was quantified using the normalized per-frame pixel difference resulting a mean motion score of 1.31 with an interquartile range (IQR) of 0.48–1.77. The median motion score has been used to distinguish recordings between low and high-motion conditions. During low-motion scenarios, the proposed framework achieved an MAE of 1.34 BPM while during high-motion conditions the corresponding MAE of 1.54 BPM respectively. These results indicate that the proposed framework maintained stable HR estimation across all the motion levels.

### 3.2 Temporal signal characteristics across facial regions

**Figure 2b** shows the temporal rPPG signals extracted from different facial sub ROIs along with their corresponding peak detection analysis. **Figure 2b** indicates that the waveform obtained from the forehead region exhibits a stable and well-defined pulsatile pattern, where the detected peaks align consistently with the underlying physiological signal. The amplitude variation remains relatively controlled over the period of time, indicating that the forehead region provides a stable and less noise-effected region for rPPG signal extraction. In contrast, the physiological waveform derived from the cheek regions demonstrates almost similar periodic structure, but with comparatively higher variability in normalized signal amplitude. Partial occasional fluctuations and transient distortions are visible in the figure which primarily reflected due to increased susceptibility to motion artifacts and illumination variations. The cheek ROIs have greater noise levels due to lighting aberrations caused by facial curvature, resulting in shadows and specular reflections under non-uniform illumination. Overall, the forehead region offers more stable pulse-related signal characteristics whereas the cheek regions retain reliable physiological information indicating that multi-region signal integration is useful to enhance robustness of the temporal signal against localized noise and motion artifacts in unconstrained environments.

### 3.3 Signal enhancement and scalogram analysis

**Figure 3:**
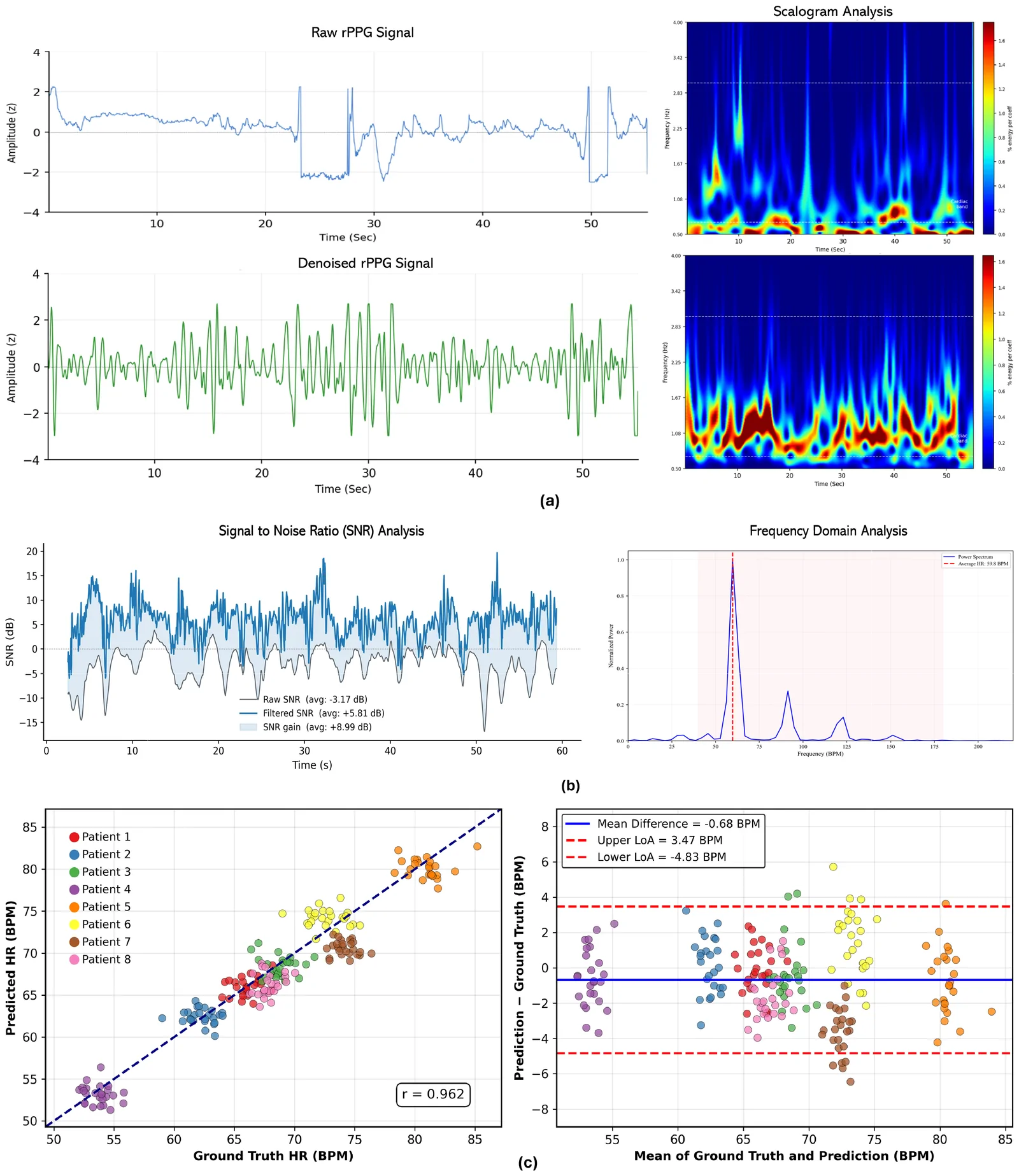
SNR enhancement and scalogram analysis **(a)** time domain and scalogram analysis **(b)** signal-to-noise (SNR) enhancement and frequency domain analysis **(c)** scatter plot and bland-altman analysis.

**Figure 3a** highlights the impact of the proposed multi-method ensemble fusion and signal processing pipeline on the raw and denoised rPPG signal through time domain analysis and scalogram analysis. **Figure 3a** indicates that the extracted raw signal components are affected by baseline drift and sudden noise fluctuations such as transient spikes, which influence the underlying physiological signal components. Through the proposed adaptive filtering stages, the raw signal is progressively refined as shown in **Figure 3a**. The resulting denoised rPPG waveform exhibits a more stable and periodic features indicating that the lowfrequency drift and high-frequency noise are removed effectively. This improvement is further reflected in the corresponding scalogram analysis where the raw signal exhibits spectral energy across a wide frequency range by reflecting the presence of non-physiological signal components. Therefore, through adaptive filtering the denoised signal’s spectral energy becomes more concentrated within the expected cardiac frequency band with clearer and consistent temporal localization. It demonstrates that the proposed framework reduces noise and enhance the interpretability of the dominant pulse-related signal components effec.

As shown in **Figure 3b**, the SNR enhancement analysis shows that the filtered signal exhibited higher average SNR values with +5.8dB than the raw signal, which exhibited SNR values close to -3.17dB. Average SNR gain is closer to + 8.99dB which confirms that the proposed signal preprocessing pipeline effectively enhances overall signal quality. **Figure 3b** shows the corresponding frequency-domain analysis of the denoised rPPG signal using power spectral density (PSD) analysis. It shows a more structured and temporally consistent distribution of energy within the cardiac band. It aligns with the observed SNR enhancement, where noise-dominated components are reduced as the overall signal becomes more stable over the processing period. So, the scalogram and PSD analysis demonstrates that the proposed pipeline enhances rPPG signal quality in both the time and frequency domains and preserves the dominant physiological characteristics required for reliable HR estimation.

**Figure 3c** presents the scatter plot and Bland-Altman agreement analysis between the proposed framework and the reference GT values across all eight patients. The Bland–Altman analysis was performed using all valid paired rPPG–ECG estimates retained after signal-quality filtering.The scatter plot shows strong correlation coefficient value of r = 0.962, which indicates a high degree of linear agreement with the ECG reference values. The Bland-Altman plot analysis shows a mean bias of −0.68 BPM, indicating slight underestimation of HR estimates in few cases with the patients. The 95% limits of agreement (LoA) ranging from −4.83 to +3.47 BPM. The corresponding 95% confidence intervals (CI) for the lower LoA and upper LoA were −5.40 to −4.26 BPM and +2.96 to +4.04 BPM, respectively. It indicates good agreement between the proposed framework and the reference values. Overall, these analyses confirm that the proposed framework achieves accurate and reliable HR estimation in real-world conditions showing robust performance in continuous physiological signal monitoring of patients in clinical environments.

## 4 Discussion and conclusion

This study investigates the feasibility of remote HR monitoring in stage-5 chronic kidney disease (CKD) patients during hemodialysis session using visible imaging and rPPG method. Proposed framework exhibits lower error metrics while compared to the ground-truth (GT) reference values. The multi-stage signal processing pipeline reduced the effects of motion artifacts, illumination variability, partial facial occlusions, and reduced face visibility. The proposed framework mainly focuses on the forehead and cheek regions which remain informative while wearing masks that partially covers the lower face area. Skin masking and anatomically defined forehead and cheek sub-ROIs further improved signal quality by focusing on physiologically informative facial regions. Proposed multi-method ensemble fusion strategy combined spectral, peak interval, and harmonic product spectrum (HPS) analyses to improve robustness, while adaptive confidence weighting reduced the influence of low-quality signal segments. It leverages complementary signal representations from multiple sources by reducing the dependency on a single method. The proposed framework provides stable heart rate estimates throughout prolonged recording sessions by down-weighting low-quality signal segments through adaptive fusion and continuous calibration. However, there are still few limitations which needs to be addressed in this study. The proposed framework was evaluated only for remote HR estimation and require further validation in cases of arrhythmia detection, intradialytic hypotension, cardiovascular events, or clinical deterioration. Future studies should validate the proposed framework in larger and more diverse patient cohorts and evaluate its performance under broader clinical conditions and multimodal sensing environments. In conclusion, this pilot study suggests that rPPG-based HR monitoring may be feasible during hemodialysis under a fixed-camera clinical setup. The proposed framework provides a baseline for remote HR monitoring in controlled clinical environments while further validation is required for broader clinical application.

## Data Availability

Data supporting the findings of this study are available from the corresponding author upon reasonable request.
